# Ultrasound Sensing Using Packaged Microsphere Cavity in the Underwater Environment

**DOI:** 10.3390/s22114190

**Published:** 2022-05-31

**Authors:** Kai Wang, Heng Wang, Xing-Yu Wu, Yong Zhang, Daquan Yang, Rongzhen Jiao, Chuan Wang

**Affiliations:** 1 School of Science, Beijing University of Posts and Telecommunications, Beijing 100876, China; wkai@bupt.edu.cn (K.W.); wangheng@bupt.edu.cn (H.W.); xingyuwu@bupt.edu.cn (X.-Y.W.); zhyong98@gmail.com (Y.Z.); rzjiao@bupt.edu.cn (R.J.); 2 The State Key Laboratory of Information Photonics and Optical Communications, Beijing University of Posts and Telecommunications, Beijing 100876, China; 3 School of Information and Communication Engineering, Beijing University of Posts and Telecommunications, Beijing 100876, China; ydq@bupt.edu.cn; 4 School of Artificial Intelligence, Beijing Normal University, Beijing 100875, China

**Keywords:** microcavity, ultrasound sensing, underwater

## Abstract

The technologies of ultrasound detection have a wide range of applications in marine science and industrial manufacturing. With the variation of the environment, the requirements of anti-interference, miniaturization, and ultra-sensitivity are put forward. Optical microcavities are often carefully designed for a variety of ultra-sensitive detections. Using the packaged microsphere cavity, we fabricated an ultrasound sensor that can work in an underwater environment. During practical detection, the optical resonance mode of the cavity can work with real-time response accordingly. The designed structure can work in various complex environments and has advantages in the fields of precision measurement and nano-particle detection.

## 1. Introduction

The rational use of ultrasound has promoted the development of modern industry and medicine. At present, it has a wide range of research and mature applications in medical imaging, marine science, industrial manufacturing, and other fields [1,2,3,4,5]. However, in some complex environments, there are several key limitations in existing ultrasound detectors. Compared with the traditional detection method using piezoelectric signals [6,7,8], the optical detection method exhibits the characteristics of anti-electromagnetic interference, and still has high sensitivity in the process of device miniaturization [9]. On the other hand, the optical whispering-gallery-mode microcavity with a small mode volume can enhance light–matter interactions. Therefore, it is often used for ultra-sensitive detection, such as particle sensing [10,11,12,13], temperature measurement [14], magnetic field sensing [15,16] and microwave frequency measurement [17].

Currently, there are many approaches to acoustic sensing based on the whispering gallery mode resonators. Most of the works use on-chip devices, such as micro-ring resonators, as sensing elements [18,19,20,21]. Admittedly, it has great advantages in certain circumstances. Considering the complex technology and manufacturing cost, some researchers turn to microbubble resonators [22,23] and microsphere resonators [24,25,26]. The hollow structure of the microbubble cavity makes it extremely sensitive to acoustic vibration signals. In the air, its noise equivalent pressure can be as low as 4.4 mPa/Hz${}^{1/2}$ [23]. On the other hand, the microsphere cavity is often used in basic research because of its simple fabrication, low cost, and high quality [27,28]. Recently, Li et al. designed a compact and highly sensitive voice-eavesdropping microresonator [26], which is used as the sensing element. Combining the high optical sensitivity of the microsphere cavity and the mechanical sensitivity of the cantilever of the microsphere cavity, the noise equivalent pressure is as low as 52 μPa/Hz${}^{1/2}$. These new structure sensors can resist electromagnetic interference and have high sensitivity. In most cases, however, they are only suitable for environments where the air is the acoustic propagation medium due to the fragile coupling. Therefore, the working environment of the sensing device will be limited.

In this paper, we fabricate an ultrasound sensor based on silica microcavities that can work in the underwater environment. The microsphere resonator and the fiber taper are packaged together, which can make the entire system maintain long-term stability. By monitoring the optical resonance mode, the ultrasonic signal applied to the test environment can be detected. In addition, we found that the resonance mode of the high-quality factor is more easily affected by ultrasound, and the high-frequency ultrasound is more easily detected. Here, the designed device has the advantages of being low-cost, easy to prepare, and recyclable. More importantly, it can break the restrictions of the working environment, which is expected to make it applicable to industrial manufacturing and vibration detection.

## 2. Experiment Methods

### 2.1. Fabrication of Microsphere Ultrasound Sensors

**Figure 1:**
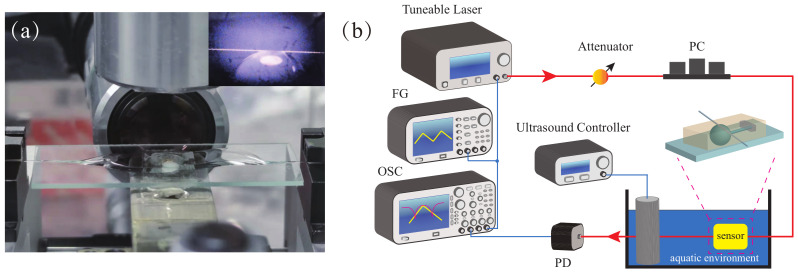
(**a**) Photograph showing the microsphere after encapsulation. Inset: incompletely packaged microsphere. (**b**) Experimental scheme of the ultrasound detection. PC, polarization controller; PD, photodetector; OSC, oscilloscope; FG, function generator.

**Figure 2:**
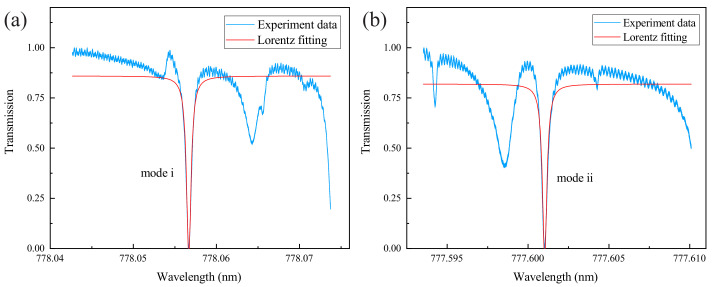
Transmission spectrum of the microsphere cavity. (**a**) Mode i with a central wavelength of 778.0567 nm. (**b**) Mode ii with a central wavelength of 777.6010 nm. The blue solid line represents the experiment data, and the red solid line represents the corresponding Lorenz fitting.

Here we prepare the microsphere by using the single-mode fibers (YOFC CS780_125-14/250) with an operating wavelength of 780 nm. Its fiber core and fiber cladding diameter are 4.2 and 125 μm, respectively. The fabrication process is as follows: Firstly, the coating at the end of the single-mode fiber is stripped off. Then, the bare fiber tip is cleaned with anhydrous ethanol. After that, a carbon dioxide laser is used to fuse the end of the fiber. Finally, a high-quality factor silica microsphere cavity can be fabricated. Since the fiber cladding diameter is 125 μm, the diameter of the microsphere cavity we prepared in this experiment is about 200 μm, and the quality factor can easily reach $10^{8}$. In particular, the ultrasound sensor device we designed is based on the change of the optical resonance mode of the microsphere cavity, which is often realized by the fiber taper in the experiment. However, if the entire system is not packaged, the coupling process between the fiber taper and the microsphere cavity cannot be maintained as stable for a long time in the underwater environment. We use optical coating material (Mypolymers MY-133-MC) to fix the coupling points and also to protect the tapered area of the fiber. The entire system is encapsulated on a glass slide, as shown in Figure 1a. Compared with the quality factor of $10^{8}$ before encapsulation, although this method sacrifices the high-quality factor of the microsphere cavity to a certain extent, it enables the system to work in a complex environment. We prepared a microsphere with a diameter of 200 μm for measurement. Benefiting from the ultra-low optical transmission loss of the microsphere cavity, its quality factor after encapsulation is still around $10^{6}$, as shown in Figure 2. Further, in Table 1, we compare the quality factors of the whispering gallery mode microcavity before and after the encapsulation in recent years.

### 2.2. Experimental Setup

The optical microcavity shows high sensitivity and slight mechanical vibration, which affects its optical resonant mode. Therefore, we designed a measurement system for sensing, as shown in Figure 1b. Considering the transmission loss of the communication band in the underwater environment, we use a tunable laser (NewFocus TLB-6712) with an optical wavelength near 780 nm. The laser is passed through an attenuator, a polarization controller, and then coupled into the microsphere cavity through a fiber taper. Finally, an oscilloscope (Tektronix MDO3104) is used for measurement after the optical signal is converted to the electrical signal by a photodetector (Newport 1801-FC). Simultaneously, the function generator (Tektronix AFG3022C) generates a triangular wave with a frequency of 50 Hz for tuning the pump wavelength and scanning the optical resonance mode in the microsphere. The two ultrasound generators (FUYANG F-103) used in the experiment have fixed frequencies, which are 26.9 and 39.4 kHz, respectively. Furthermore, the intensity of ultrasound signals can be tuned by tuning the current.

In order to measure the response of the microsphere resonant modes to the ultrasound signals, we pre-select two resonant modes with high-quality factors, as shown in Figure 2. The resonant wavelengths of mode i and ii are 778.0567 and 777.6010 nm, respectively. The quality factors of the modes are $Q_{i}=1.2\times 10^{6}$ and $Q_{ii}=2.3\times 10^{6}$, respectively. Taking mode i as an example, first, an ultrasound source with a frequency of 26.9 kHz is placed in water, and then different ultrasound powers are selected to measure the response of mode i. Note that it is necessary to turn off the ultrasound source whenever changing the ultrasound power and wait for the mode i to return to the original position before measuring. This is to ensure that the measurement process is in the same measurement environment. After that, on the premise of keeping the original optical mode i unchanged, the ultrasound source with a frequency of 39.4 kHz is replaced to repeat the above measurement process. Since the thermal effect of the microcavity [33] is unfavorable for this experiment, it is necessary to adjust the optical signal input to below the threshold power using an attenuator.

## 3. Phenomenological Theoretical Model

In this section, we propose a phenomenological theoretical model of the sensor system. First, we focus on the absence of an ultrasound signal in the environment. In this case, a single optical resonance mode is described by the well-known coupled-mode theory [34]. The transmission spectrum of the system could be written as

(1) $$T\left(\omega \right)=\frac{\Delta^{2}+{\left(\kappa_{0}-\kappa_{ex}\right)}^{2}/4}{\Delta^{2}+{\left(\kappa_{0}+\kappa_{ex}\right)}^{2}/4}.$$

Here, $\Delta =\omega -\omega_{c}$ is the detuning of the laser. $\omega_{c}$ is the central resonant frequency of the cavity mode. $\kappa_{0}$ and $\kappa_{ex}$ are the intrinsic dissipation and the coupling dissipation, respectively. When the intrinsic dissipation equals the coupled dissipation, the system approaches the critical coupling condition. However, when the ultrasound source is turned on, the mechanical vibrations generated by the ultrasound will cause the optical coating material to be squeezed. Its interior undergoes localized deformation, changing its surrounding density and refractive index. Simultaneously, the evanescent fields of the whispering gallery mode and the fiber taper are inside the optical coating material. Therefore, when the refractive index of the optical coating material changes, the resonance conditions of the microsphere cavity are also affected. This will alter the resonance wavelength and mode linewidth. Zhu et al. have fabricated a magnetometer using a similar sensing mechanism [35]. Furthermore, we phenomenologically attach this change in refractive index to $\omega_{u}$ and $\kappa_{u}$ and add them to Equation (1). In this way, the transmission spectrum of the system can be described as

(2) $$T_{u}\left(\omega \right)=\frac{\Delta_{u}^{2}+{\left[\kappa_{0}-\left(\kappa_{ex}+\kappa_{u}\right)\right]}^{2}/4}{\Delta_{u}^{2}+{\left[\kappa_{0}+\left(\kappa_{ex}+\kappa_{u}\right)\right]}^{2}/4}.$$

**Figure 3:**
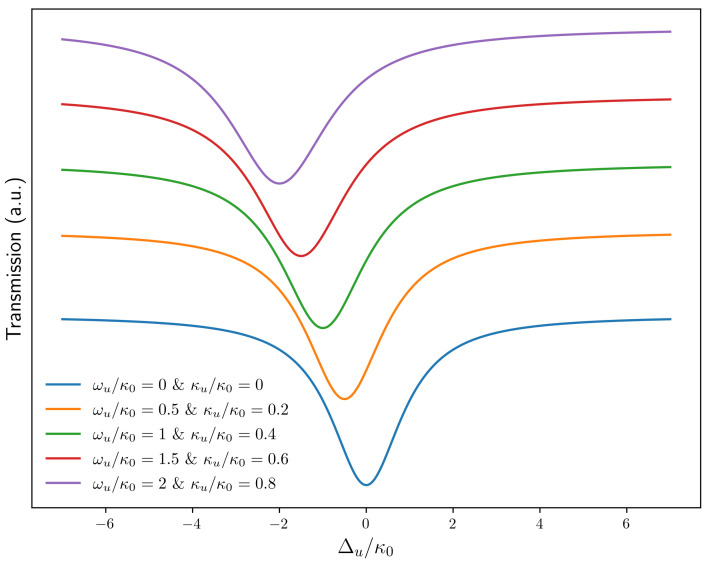
The numerical simulation of the changing of the transmission spectrum of the system in the presence of ultrasound.

Here, $\Delta_{u}=\omega -\omega_{c^{{}^{\prime }}}$ is the detuning of the input field after ultrasound tuning. $\omega_{c^{{}^{\prime }}}=\omega_{c}-\omega_{u}$ denotes the resonance frequency after ultrasound tuning. $\kappa_{u}$ is the additional coupling dissipation due to the ultrasound source. The change in transmission spectrum is numerically simulated in Figure 3. Due to the limitations of the ultrasound device we are using, it is hard to measure a wider range of ultrasound frequencies. Therefore, the change in transmission spectrum can only be simulated phenomenologically.

## 4. Results and Discussion

**Figure 4:**
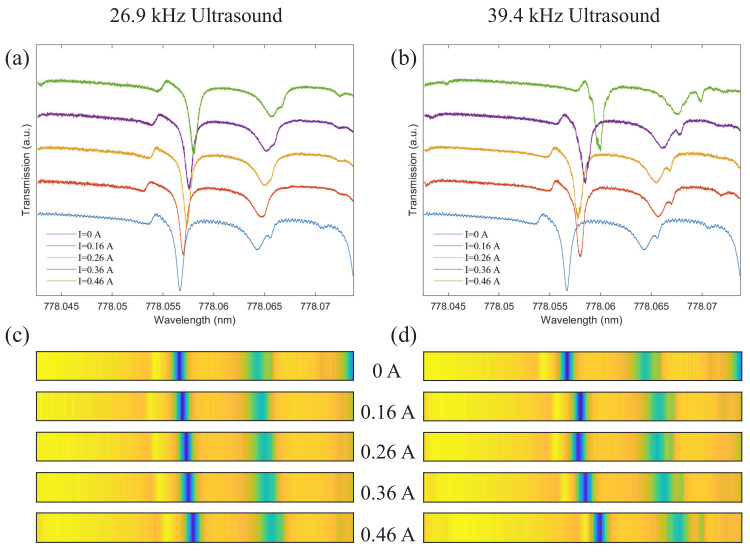
Variation trend of mode i with ultrasound. (**a**) The response of mode i to the 26.9 kHz ultrasound. (**b**) The response of mode i to the 39.4 kHz ultrasound. (**c**) Optical barcode corresponding to (**a**). (**d**) Optical barcode corresponding to (**b**).

To demonstrate the principle of ultrasound sensing, the response of mode i to different ultrasound intensities is measured at ultrasound frequencies of 26.9 and 39.4 kHz, respectively, as shown in Figure 4. It can be seen that along with the increase in ultrasound intensity, the optical resonance mode of the microsphere is obviously shifted. As we mentioned in Section 3, different intensities of ultrasound generate different magnitudes of sound pressure, which lead to different local deformation of the optical coating material. This ultimately alters its refractive index, causing a frequency shift of the optical resonance mode. By comparing Figure 4a,b, we find that the shift of mode i is larger when the ultrasound frequency is 39.4 kHz under the same environment. That is to say, under the same circumstances, the stronger the ultrasound frequency, the more obvious the local deformation of the optical coating material, and the greater the change of the refractive index. It can be determined that the change in the optical resonance mode is determined by the combined effect of ultrasound frequency and intensity. Moreover, optical barcodes of cavity modes are more useful for describing specific variations of multiple optical resonance modes. It is widely used in the research of precise temperature measurement [14] and microwave frequency measurement [17]. The optical barcodes corresponding to the two ultrasound frequencies are shown in Figure 4c,d, respectively.

**Figure 5:**
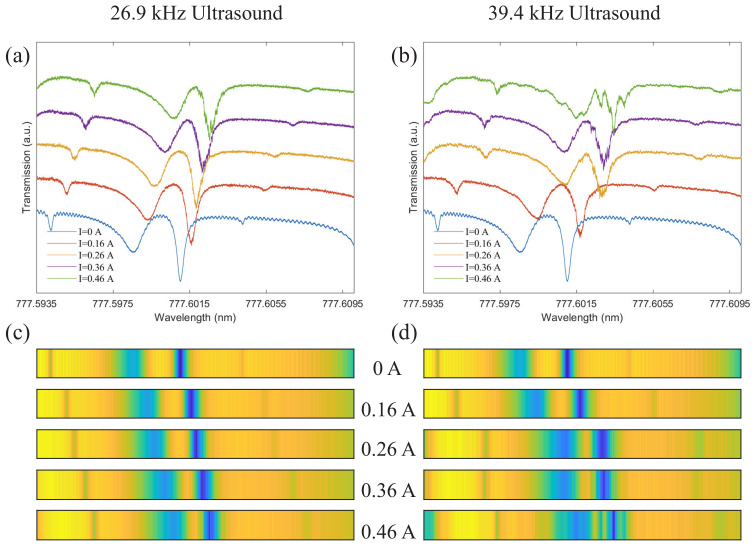
Variation trend of the resonance of mode ii with ultrasound. (**a**) The response of mode ii to the 26.9 kHz ultrasound. (**b**) The response of mode ii to the 39.4 kHz ultrasound. (**c**) Optical barcode corresponding to (**a**). (**d**) Optical barcode corresponding to (**b**).

**Figure 6:**
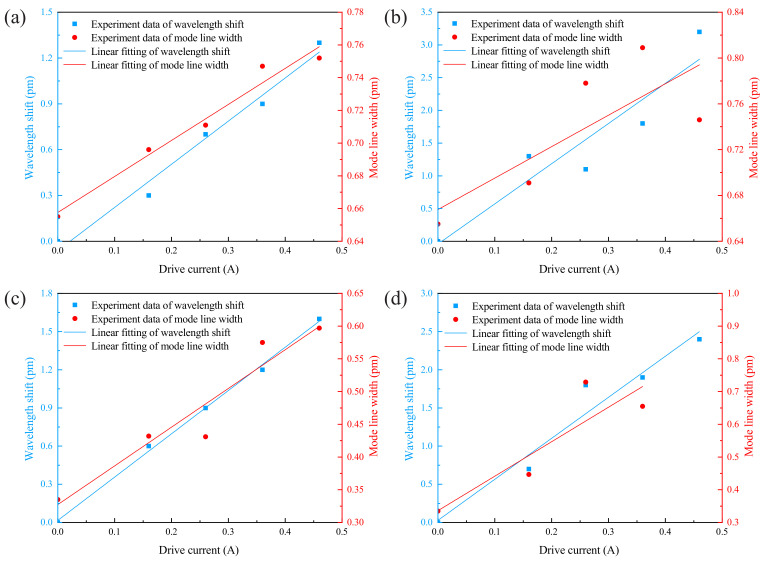
Experimental data and fitted curves of resonance wavelength shift and mode linewidth. (**a**) Mode i under the 26.9 kHz ultrasound. (**b**) Mode i under the 39.4 kHz ultrasound. (**c**) Mode ii under the 26.9 kHz ultrasound. (**d**) Mode ii under the 39.4 kHz ultrasound.

Notably, a key factor for an optical microcavity to act as a high-sensitivity sensing device is its high-quality factor. To this end, we measured the response of another mode ii with a high-quality factor to ultrasound on the basis of the above studies, and the results are shown in Figure 5. It can be seen intuitively that the modulation of the high-quality factor of the optical resonance mode spectrum by ultrasound is obvious. To further study the changes in optical resonant modes, we extracted the wavelength shifts and mode linewidths under each of the above experimental conditions, as shown in Figure 6. For mode i, when the ultrasound frequencies are 26.9 and 39.4 kHz, the slopes of the wavelength shift after linear fitting are $k_{i-wl1}=2.83$ and $k_{i-wl2}=6.138$, respectively; and the slopes of the mode linewidth are $k_{i-mlw1}=0.22$ and $k_{i-mlw2}=0.275$, respectively. For mode ii, when the ultrasound frequencies are 26.9 and 39.4 kHz, the slopes of the wavelength shift are $k_{ii-wl1}=3.4$, $k_{ii-wl2}=5.37$, respectively; and the slopes of the mode linewidth are $k_{ii-mlw1}=0.59$ and $k_{ii-mlw2}=1.05$, respectively. From the fitting results, the responses of different modes to ultrasound with the same frequency and intensity are slightly different. If only the change of a single resonant mode is considered, there will be a large error. However, the use of optical barcodes with multiple resonance modes can reduce measurement errors to some extent. On the other hand, as shown in Figure 6d, when fitting the mode linewidth, we drop the last point. This is because the resonance mode is modulated by high-frequency and high-intensity ultrasound, which affects the mode linewidth and leads to inaccurate fitting.

## 5. Conclusions

In summary, we experimentally realized an ultrasound detection device that can work in the underwater environment. The unique design features, packaged microsphere cavity and fiber tapered waveguide of the ultrasound detection device permit the high-quality factor ($10^{6}$) to be maintained in an aqueous environment. The presence of the ultrasound signals can be judged by monitoring the optical resonance mode of the microsphere cavity. Simultaneously, the higher the quality factor, the more obvious the phenomenon of ultrasound modulation. When the whole system is at the same ultrasound frequency, as the driving current increases, the optical resonance wavelength will be red-shifted, and the linewidth of the mode will also be broadened. On the other hand, when the whole system is under the same driving intensity, higher frequency ultrasound will lead to a more obvious mode redshift. Since each optical mode has different response conditions, this method has limitations for the direct measurement of ultrasound frequency and intensity. In order to obtain more accurate results, optical barcodes with multiple optical modes can be used for characterization. More interestingly, the coupling system can be packaged into different devices to measure weak vibration signals for more complex environments.

## Figures and Tables

**Table 1:** Variation found in the literature on the quality factor of whispering gallery mode microcavities before and after encapsulation.

Reference	Diameter	Before Encapsulation	After Encapsulation
F. Monifi, et al. (2013) [29]	120 μm	$5\times 10^{7}$	$1\times 10^{7}$
Wang, et al. (2014) [30]	153 μm	−	$0.9\times 10^{5}$
Zhao, et al. (2017) [31]	80 μm	−	$2\times 10^{7}$
Yang, et al. (2020) [32]	90 μm	$10^{7}$	$3.52\times 10^{6}$
Sun, et al. (2021) [25]	60 μm	−	$8.5\times 10^{5}$

## Data Availability

Not applicable.
